# High frequency of hybrid *Escherichia coli* strains with combined Intestinal Pathogenic *Escherichia coli* (IPEC) and Extraintestinal Pathogenic *Escherichia coli* (ExPEC) virulence factors isolated from human faecal samples

**DOI:** 10.1186/s12879-018-3449-2

**Published:** 2018-11-01

**Authors:** Bjørn-Arne Lindstedt, Misti D. Finton, Davide Porcellato, Lin T. Brandal

**Affiliations:** 1 Faculty of Chemistry, Biotechnology and Food Science, Norwegian University of Life Sciences, P.O. Box 5003, N-1432 Ås, Norway; 2 Department of Zoonotic, Food- and Waterborne Infections, Norwegian Institute of Public Health, Oslo, Norway

**Keywords:** *Escherichia coli*, Pathogenic, ExPEC, IPEC, Hybrid strains, MinION

## Abstract

**Background:**

Classification of pathogenic *Escherichia coli* (*E. coli*) has traditionally relied on detecting specific virulence associated genes (VAGs) or combinations thereof. For *E. coli* isolated from faecal samples, the presence of specific genes associated with different intestinal pathogenic pathovars will determine their classification and further course of action. However, the *E. coli* genome is not a static entity, and hybrid strains are emerging that cross the pathovar definitions. Hybrid strains may show gene contents previously associated with several distinct pathovars making the correct diagnostic classification difficult. We extended the analysis of routinely submitted faecal isolates to include known virulence associated genes that are usually not examined in faecal isolates to detect the frequency of possible hybrid strains.

**Methods:**

From September 2012 to February 2013, 168 faecal isolates of *E. coli* routinely submitted to the Norwegian Institute of Public Health (NIPH) from clinical microbiological laboratories throughout Norway were analysed for 33 VAGs using multiplex-PCR, including factors associated with extraintestinal pathogenic *E. coli* (ExPEC) strains. The strains were further typed by Multiple Locus Variable-Number Tandem-Repeat Analysis (MLVA), and the phylogenetic grouping was determined. One isolate from the study was selected for whole genome sequencing (WGS) with a combination of Oxford Nanopore’s MinION and Illumina’s MiSeq.

**Results:**

The analysis showed a surprisingly high number of strains carrying ExPEC associated VAGs and strains carrying a combination of both intestinal pathogenic *E. coli* (IPEC) and ExPEC VAGs. In particular, 93.5% (101/108) of isolates classified as belonging to an IPEC pathovar additionally carried ExPEC VAGs. WGS analysis of a selected hybrid strain revealed that it could, with present classification criteria, be classified as belonging to all of the Enteropathogenic *Escherichia coli* (EPEC), Uropathogenic *Escherichia coli* (UPEC), Neonatal meningitis *Escherichia coli* (NMEC) and Avian pathogenic *Escherichia coli* (APEC) pathovars.

**Conclusion:**

Hybrid ExPEC/IPEC *E. coli* strains were found at a very high frequency in faecal samples and were in fact the predominant species present. A sequenced hybrid isolate was confirmed to be a cross-pathovar strain possessing recognised hallmarks of several pathovars, and a genome heavily influenced by horizontal gene transfer.

**Electronic supplementary material:**

The online version of this article (10.1186/s12879-018-3449-2) contains supplementary material, which is available to authorized users.

## Background

*Escherichia coli* (*E. coli*) is a highly diverse and predominant species among facultative anaerobic bacteria of the human gastrointestinal tract [1]. *E. coli* comprises non-pathogenic commensals as well as strains causing a range of diseases. *E. coli* strains capable of causing extraintestinal infections are designated as extraintestinal pathogenic *E. coli* (ExPEC) to distinguish them from strains causing intestinal disease, commonly designated as intestinal pathogenic *E. coli* (IPEC).

ExPEC can cause a wide variety of extraintestinal infections at multiple anatomical sites. ExPEC frequently cause urinary tract infection (UTI), septicaemia, meningitis, as well as causing soft tissue damage [2, 3]. ExPEC includes, among others, the pathovars uropathogenic *E. coli* (UPEC) associated with urinary tract infection in human and animals, neonatal meningitis-associated *E. coli* (NMEC), septicaemic *E. coli* (SePEC) causing systemic infection in human and animals, avian pathogenic *E. coli* (APEC) that cause avian colibacillosis, and a potentially emerging ExPEC lineage named endometrial pathogenic *E. coli* (EnPEC) [4, 5]*.*

A wide range of VAGs have been associated with ExPEC and common virulence attributes among ExPEC strains are those enabling their extraintestinal lifestyle e.g. genes coding for the production of adhesins, toxins, protectins, siderophores, iron transport systems, and invasins [2, 6–9]. It is believed that ExPEC are facultative pathogens, which reside in the normal gut flora as commensals in some groups of the healthy population [8]. However, there are no universal accepted concrete genetic criteria for defining an *E. coli* strain as ExPEC nor for definite pathovar classification within the ExPEC group. Thus, the true pathovar classification can only be done on the basis of the isolation source for the majority of ExPECs.

There is limited information regarding the frequency of ExPEC strains in the human intestine, however a recent meta study of more than 500 published papers assessed a prevalence of ExPEC strains among faecal isolates of about 10% in healthy individuals [10]. Reference laboratories or diagnostic microbiological laboratories routinely search for only the established IPEC virulence factors in faecal samples from symptomatic patients. There exist little data on the frequency of ExPEC related virulence factors among these strains.

The aim of this study was to investigate the frequency and combination of virulence markers including VAGs used for IPEC pathovar classification and a selection of VAGs related to ExPEC pathovars among *E. coli* strains submitted from individuals showing signs of gastrointestinal infections. We assessed the frequency of ExPEC and IPEC strains, phylogenetic grouping and the MLVA-genotype.

In light of the large German O104:H4 outbreak in 2011 [11], which was caused by a hybrid Enteroaggregative *E. coli* (EAEC)/Shiga toxin producing *E. coli* (STEC) strain [12], the monitoring of isolates to detect new or altered combinations of VAGs is important as it may give a pre-warning of emerging strains harbouring novel VAG combinations, which should be studied in closer detail to assess whether they also have altered virulence capabilities.

## Methods

### Bacterial isolates

All 168 *E.coli* strains were obtained from the culture collection at the National Reference Laboratory for Enteropathogenic Bacteria at the Norwegian Institute of Public Health (NIPH).

### VAG PCR

#### *ExPEC VAGs, cnf1, cnf2, cnf3, ehaA* and *ehaG* PCR

PCR-primers for amplication of the following VAGs were constructed using primer3 software (http://www.ncbi.nlm.nih.gov/tools/primer-blast/) and DNASTAR’s Lasergene software module “Primer Select” (DNAstar, Inc., Madison, WI): cytotoxic necrotising factors 1–3 *cnf1, cnf2, cnf3*; autotransporters (ATs) *sat, tsh, vat, ehaA,* and *ehaG*; iron acquisition *iutA*, *sitA, iucD, iroC, fbpB,* and *fyuA;* adhesins *sfaS, papC,* and *tosA*; protectins *kpsS, traT* and *iss*; the invasin gene *ibeA,* and primers directed at orf5 in the gimB genetic island (sequence acc. no. AY170898). Primers directed at the *etsA* gene encoding the macrolide-specific efflux protein EtsA were also designed (see Additional file 1). PCR primers aimed at ExPEC VAGs and ehaA + ehaG were combined in four multiplex reaction mixes as follows: Multiplex 1 (*cnf1, cnf2, cnf3, iutA, ibeA and sitA*), Multiplex 2 (*iucD, iss, traT, iroC, sat, papC and ehaA*), Multiplex 3 (*tsh*, gimB-genetic island, *etsA, kpsS* and *sfaS*), and Multiplex 4 (*tosA, vat, fbpB, fyuA* and *ehaG*).

All primers had a final concentration of 5 μM. The PCR was run on a GeneAmp 9700 thermocycler (Applied-Biosystems, Foster City, CA, USA) with the following conditions: multiplexes 1, 2 and 4; 95 °C for 15 min, then 25 cycles of 94 °C for 30 s, 58 °C for 90 s and 72 °C for 90 s, followed by a hold on 72 °C for 10 min after temperature cycling has ended. Multiplex 3; 95 °C for 15 min, then 25 cycles of 94 °C for 30 s, 60 °C for 90 s and 72 °C for 90 s, followed by a hold on 72 °C for 10 min after temperature cycling has ended. The multiplexes were diluted 1:25 and run in separate capillaries on an ABI 3130 Genetic Analyzer (Applied-Biosystems, Foster City, CA, USA) with GS 600LIZ as internal size standard.

#### IPEC VAGs

PCR for detecting common IPEC VAGs was performed as previously published [13–15]. In all, primers for the following IPEC VAGs were included: *stx1, stx2, eaeA, ipaH*, *LTI, STIa, STIb, aggR, ehxA, bfp* with 16S control *rrs* (see Additional file 1).

### Phylogenetic group PCR

The improved phylogenetic PCR-assay [16] of the original assay described by Clermont [17] was used to assign the *E. coli* isolates to major phylogenetic groups and subgroups.

### MLVA

Multi-locus variable-number tandem repeats analysis was performed using a modified version of the 10-loci generic *E. coli* MLVA scheme previously published [18]. The PCR-amplicon of the published CCR001 locus contains two variable repeated elements, and the modified scheme allows typing of both these variable elements increasing the number of the generic *E. coli* MLVA to 11-loci. The modification consists of a change of dyes and an additional new reverse-primer at the CCR001 locus as follows: the 6FAM dye was removed from the published CCR001 forward primer [18] and the published unlabelled CCR001 reverse primer was labelled with 6FAM and renamed CCR001aR. A new second VIC-labelled reverse primer was added “CCR001bR: 5’ - VIC-CGCATTTTATCTGTCTGTACGGC – 3’”. The combination of both reverse primers made it possible to simultaneously separate both repeat containing regions at the CCR001 locus.

### Stx subtyping

Subtyping of *stx1* and *stx2* was performed as described in Brandal et al. 2015 [15].

### Oxford Nanopore MinION sequencing

The hybrid ExPEC/IPEC strain FHI_NMBU_03 identified by PCR, was chosen for sequencing by the MinION MK1 device. DNA was quantified using the Qubit fluorometer (Life Technologies, Paisley, UK) and 200 ng of DNA was used for library preparation. The strain was sequenced using the R9.4 SpotON flow cell and the SQK-RAD002 rapid sequencing kit. All runs were prepared according to the standard protocol of Oxford Nanopore Technologies (Oxford, UK). The flow cells were primed with a priming solution that consisted of a mixture of nuclease free water and Fuel Mix. The library was then loaded into the MinION SpotON port and the 48-h sequencing protocol was selected in the MinKNOW software. The basecalling was done through the Metrichor Desktop Agent using 1D Basecalling for the SQK-RAD002 protocol.

### Illumina MiSeq sequencing

Illumina sequencing was performed on an Illumina MiSeq platform (Illumina Inc., San Diego, CA, USA). Library was prepared using the Nextera XT kit (Illumina Inc) according to manufacturer’s instructions and was sequenced using a 300 bp paired-end sequencing kit (Illumina Inc).

### Sequence analysis

Raw Illumina reads were paired and quality filtered using Trimmomatic [19] and bases with low quality (< q20) were discarded. MinION reads were extracted using poRe [20] and both read types were assembled using SPAdes [21] version 3.5.0 using the option “--nanopore”.

Using combined MiSeq and MinION data, the sequences were assembled into a large contig constituting the genome and a contig containing a large virulence plasmid.

The sequence data was annotated using four different services, the NCBI Prokaryotic Genome Annotation Pipeline [22], the BASys Bacterial Annotation System [23], The RAST Annotation Server [24] and Prokka [25]. The sequences were further analysed using a variety of free and publicly available software. Integrated prophages and genomic islands (GIs) were searched using PHASTER [26] and Island Viewer 4 [27] respectively, and the final location of prophages and GIs was determined using a combination of the resulting data. Multilocus sequence typing (MLST)-type, Fim-type, antibiotic resistance genes, and virulence genes were searched using online services from the Center for Genomic Epidemiology (CGE) at the Danish Technical University (DTU), Lyngby, Denmark (http://www.genomicepidemiology.org/). Assembly and annotation of the isolate FHI_NMBU_03 and its plasmid are publicly available at NCBI (accession number CP019455 and CP019456, respectively).

## Results

### PCR

The pathovar distribution among the 168 *E. coli* faecal isolates were as follows: 53 non-IPEC (31.5%), (including 2 strains harbouring *ehaG* only and 1 strain negative for all tested VAGs). One hundred eight IPEC (64.3%), (including 49 atypical-EPEC (aEPEC) (29.2%), 31 STEC (18.5%), 21 enterotoxigenic *Escherichia coli* (ETEC) (12.5%), 7 necrotoxin producing *E. coli* (NTEC) (4.2%), 3 enteroinvasive *Escherichia coli* (EIEC) (1.8%), 2 EAEC (1.2%), 1 typical-EPEC (tEPEC) (0.6%), and 1 STEC/ETEC (*stx2d, LTI, iss, traT* and *ehaG*) hybrid strain (0.6%)). A total of 108 isolates (64.3%) contained both recognised IPEC and ExPEC VAGs, thus 93.9% (108/115) of the IPEC isolates also carried ExPEC VAGs. Fifty isolates (29.7%) carried only recognised ExPEC VAGs without any accompanying IPEC associated genes (Table 1). The frequency of the phylogenetic subgroups were: 15 A0 (8.9%), 35 A1 (20.8%), 64 B1 (38.1%), 7 B2_2 (4.2%), 21 B2_3 (12.5%), 20 D1 (11.9%) and 6 D2 (3.6%). The phylogenetic group distribution within each pathovar can be seen in Table 2. The highest frequency of combinatory IPEC/ExPEC strains was seen in phylogenetic subgroup B2_2 and group B1 (100 and 75%, respectively). The frequency of the tested ExPEC related VAGs among all isolates can be seen in Table 3. The *ehaG* gene was detected in 64.3% of the isolates and was the most common VAG in our collection. *eae, ehaA, ehxA* and the gimB genetic island marker were present in 44.6, 38.7, 15.5, and 1.2% of the isolates, respectively. When we looked at the average number of VAGs within all phylogenetic subgroups, we found that subgroup B2_2 carried most VAGs (7 VAGs) followed by B2_3 (6.9 VAGs), D2 (5.7 VAGs), D1 (5.4 VAGs), B1 (5.1 VAGs), A1 (4.1 VAGs), and A0 (3.3 VAGs).

**Table 1:** Distribution of pathotypes in *E. coli* faecal isolates

Pathotype	ExPEC^a^	All IPEC^b^	Other^c^	IPEC VF only^d^	IPEC/EXPEC^e^
Number	50	115	3	7	108
Percent	29.7%	68.5%	1.8%	4.2%	64.3%

^a^Number of isolates with ExPEC VAGs only

^b^Number of isolates containing an IPEC VAG

^c^Two isolates positive for the *ehaG* gene only, and one isolate negative for all 33 markers

^d^Number of isolates with IPEC VAGs exclusively

^e^Number of isolates positive for combinations of both IPEC and ExPEC VAGs

**Table 2:** Phylogenetic group distribution within each pathovar

Phylogroup	A		B1	B2		D	
Subgroup	A0	A1	B1	B2_2	B2_3	D1	D2
aEPEC	5	7	17	7	8	4	1
tEPEC	0	0	0	0	1	0	0
STEC	0	1	24	0	0	6	0
ETEC	0	8	11	0	0	1	1
EAEC	0	0	1	0	0	1	0
EIEC	2	0	1	0	0	0	0
STEC/ETEC	0	0	0	0	0	0	1
NTEC	0	1	0	0	6	0	0
NON-IPEC	8	18	10	0	6	8	3
Σ Subgroups	15	35	64	7	21	20	6
Σ Phylogroups	50 (29.8%)		64 (30.1%)	28 (16.7%)		26 (15.5%)

**Table 3:** Frequency of ExPEC associated virulence genes (PCR screening)

ExPEC associated VAG	Comment	Frequency
*iss*	Increased serum survival gene	48.8%
*traT*	Gene encoding complement resistance protein	45.2%
*fyuA*	Ferric yersiniabactin uptake receptor gene	42.3%
*iucD*	Aerobactin biosynthesis gene	24.4%
*iutA*	Ferric aerobactin receptor gene	23.8%
*sitA*	Iron/manganese transport system periplasmic binding protein gene	23.2%
*kpsS*	Capsule polysaccharide export protein gene	18.5%
*tsh*	Temperature-sensitive hemagglutinin autotransporter gene	15.5%
*iroC*	Salmochelin siderophore system gene	12.5%
*vat*	Vacuolating autotransporter toxin gene	11.3%
*fbpB*	Gene associated with urinary tract infections	10.1%
*sat*	Secreted autotransporter toxin gene	9.5%
*ibeA*	Invasion protein gene	8.3%
*etsA*	Macrolide-specific efflux protein gene	4.2%
*cnf1*	Gene encoding the cytotoxic necrotizing factor 1	3.6%
*sfaS*	S-fimbrial adhesin gene	1.8%
*papC*	P-fimbriae outer membrane usher protein gene	1.8%
*tosA*	Repeat-in-toxin gene	1.8%
*cnf2*	Gene encoding the cytotoxic necrotizing factor 2	0.6%
*cnf3*	Gene encoding the cytotoxic necrotizing factor 3	ND^a^

^a^Not detected

The 168 isolates grouped into 131 different MLVA-profiles (1.23 isolates/MLVA-profile), where six clusters of identical MLVA-profiles containing three or more isolates were detected. Cluster 1 consisted of five ExPEC isolates of phylogenetic group A1, all from December 2012. Four of the isolates shared the same VAGs (*sitA, iss, traT, kpsS* and *ehaG*), while the fifth isolate had a deviating VAG composition (*iutA, cnf2, iucD, iss, traT, ehaA, fbpB* and *ehaG*) and was designated NTEC due to the presence of the gene for cytotoxic necrotising factor 2 (*cnf2*). Cluster 2 comprised of three aEPEC strains of phylogenetic group D1 isolated in October and November 2012, all of serogroup O55 with identical VAGs (*eae, iss, ehaA, fbpB* and *ehaG*). Cluster 3 contained six phylogenetic group B1 isolates from December 2012, where five isolates shared the same VAGs (*LTI, iss, fyuA* and *ehaG*) and was designated ETEC due to the presence of the *LTI* gene. Of these five isolates, four were serotyped into serogroup O78 while no serogroup could be assigned to the fifth isolate. The sixth isolate of MLVA-cluster 3 was also an O78 B1 isolate, but with different VAGs (*sitA, iss, traT, kpsS, fyuA* and *ehaG*). Cluster 4 consisted of four phylogenetic group B1 serogroup O103 STEC isolates from September to December 2012, all with identical VAGs (*stx1a, eae, ehxA, traT, ehaA* and *ehaG*). Cluster 5 consisted of six phylogenetic group B1 serotype O103:H2 STEC isolates from October and November 2012 submitted from the same Norwegian hospital with identical VAGs (*stx1a, eae ehxA, traT, ehaA* and *ehaG*). Cluster 6 contained four phylogenetic group B2_2 aEPEC isolates from September and October 2012 where three of the isolates showed the same VAGs (*eae, ibeA, iss, traT, iroC, tsh, vat* and *fyuA*), while the fourth isolate had the following VAGs (*eae, ibeA, tsh, vat* and *fyuA*).

Among the 49 *eae* containing aEPEC isolates, the following VAGs were additionally detected: *sitA, iss, ehaA, ehaG, papC, tsh, kpsS, vat, fyuA, iutA, iucD, fbpB, ehxA, sat, tsh, traT, ibeA, iroC, etsA, tosA*, as well as a marker in the gimB genetic island. Only 4 of 49 aEPEC isolates (8.2%) did not carry any VAGs previously associated with ExPEC strains. Thus, the majority (91.8%) of our aEPEC faecal isolates contained VAGs related to ExPEC strains. The most common ExPEC related VAGs among the aEPEC isolates were: *traT* (49%), *iss* (38.8%), *fyuA* (32.7%), *tsh* (26.5%) and *ibeA* (26.5%). When we divided the aEPEC isolates by phylogenetic group, we observed that the *ibeA* gene was present in 86.7% (13/15) of the aEPEC B2 strains, and the VAGs *ehaA* and *ehaG* were also frequently present, 49 and 51% respectively.

The 31 STEC isolates contained 18 *stx1* only positive strains and 9 *stx2* only positive strains. The remaining four strains contained both *stx1* and *stx2*. Among the STEC isolates, the following VAGs were additionally found: *eae, iutA, iucD, iss, traT, iroC, ehaA, ehaG, etsA, fyuA, kpsS, ehxA* and *fbpB.* The most common ExPEC related VAGs were: *traT* (58%), *iss* (35.5%), *iucD* (29%) and *iutA* (25.8%). Additional prevalent non-ExPEC factors present were: *ehaA* (96.8%), *ehaG* (90.3%), *ehxA* (74.2%) and *eae* (71%).

Among the 21 ETEC isolates, *ehaG* was detected in 12 strains (57%), but *ehaA* was not detected in any of the ETEC isolates.

When we looked at pair-clustering of the VAGs we found that the most common pairs (in more than 20% of isolates) of VAGs included: *ehaA* and *ehaG* in 60/168 (35.7%) of the isolates, *ehaG* and *traT* or *iss* both combinations in 49/168 (29.2%) of the isolates, *eae* and *ehaA* in 48/168 (28.6%) of the isolates, *eae* and *ehaG* in 46/168 (27.3%) of the isolates, *iss* and *fyuA* in 43/168 (25.6%) of the isolates, *traT* and *eae* or *ehaA* both combinations in 40/168 (23.8%) of the isolates, *iucD* and *iutA* in 40/168 (23.8%) of the isolates and *traT* and *iss* in 38/168 (22.6%) of the isolates.

### Sequencing

One strain from this study designated FHI_NMBU_03 from MLVA-cluster 6 was selected for whole genome sequencing using a combination of long- and short- read technologies, Oxford Nanopore MinION (91,865 reads) and Illumina MiSeq (361,031 reads), respectively. We were able to assemble a complete closed circular genome (4,685,056 bp acc. nr. CP019455) and a complete circular virulence plasmid (159,821 bp acc. nr. CP019456) pFHI_NMBU_03–1 from the combined runs. The genome sequence (coverage 21.6x) contained 4954 genes (gene density 1.057 genes/Kbp) and 200 pseudogenes, with a GC content of 51%. The chromosome contains five integrated prophages according to PHASTER analysis [26], and 19 genomic islands (phages excluded) according to the Island Viewer 4 software [27]. FHI_NMBU_03 showed a surprising collection of both IPEC and ExPEC related VAGs as indicated by the PCR-analysis. It contained the locus of enterocyte effacement (LEE)-region of EPEC/EHEC as well as recognized markers for ExPEC subtypes of UPEC/APEC and NMEC. The LEE region of FHI_NMBU_03 contains 36 recognized genes, four open reading frames (ORFs) of unknown function as well as two pseudogenes, and is inserted in the *selC* tRNA gene. The *eae*-intimin subtype of FHI_NMBU_03 is *β2*. The LEE-encoded Tir protein of FHI_NMBU_03 is, by BLAST search, identical to three Tir proteins from EPEC strains and one protein from a human strain designated as UPEC (upec-202, SAMN02802023), as well as eight animal strains. Additionally the genome encodes the intimin-like proteins FdeC and a SinH-variant. FHI_NMBU_03 was also positive for a cluster of the non–LEE-encoded effectors *nleB, nleC, nleG, nleH* and a frameshifted *nleA* pseudogene, located within a phage-region identified by PHASTER. Using CGE the MLST type was predicted to be ST28 and the *fimH* subtype was predicted to fimH90. A selection of chromosomal genes found by sequencing associated with virulence can be seen in Table 4. On the large virulence plasmid, ExPEC pathogenicity associated genes include: *bor* (an *iss* homologue), *traT* (serum resistance associated)*,* the pyelonephritis-associated pilus *pap* operon; *papABCDEFHJK*, a putative *pixG* adhesin related gene encoding a protein 99% identical to a protein (EQZ28352.1) from the *E. coli* human UTI strain UMEA- 3585-1 (PRJNA186355), a putative autotransporter gene encoding an uncharacterized protein identical to protein EQZ28355.1 from UMEA- 3585-1, *iroN* (catecholate siderophore receptor), an AppA (HlyII) hemolysin protein and the leukotoxin genes *lktBCD*.

**Table 4:** Selected virulence associated genes found on the FHI-NMBU-03 chromosome by nBLAST

Gene name (FHI-NMBU-03 chromosome)	Comment	Associated pathovar	% identity	Cover.	BLAST sequence
*aatB*	Autotransporter adhesin and virulence factor of avian pathogenic *Escherichia coli.*	APEC	98.43	1017 / 1017	JX402062
*herA*	Archaeal bi-polar DNA helicase	Unknown	99.47	1686 / 1686	NZ_NLRN01000019
*aslA*	Arylsulfatase gene	ExPEC (Invasive K1 strains)	98.55	1656 / 1656	CU928163
*aufC*	Fimbrial usher protein gene	UPEC	99.50	2595 / 2595	KE702411
*cesAB*	Enteropathogenic *Escherichia coli* chaperone for the type-III translocator proteins	EPEC/STEC	100.00	324 / 324	FM986651
*cesD2*	A second chaperone for the type III secretion translocator protein EspD	EPEC/STEC	98.28	407 / 408	NC013364
*cesT*	A bivalent enteropathogenic Escherichia coli chaperone required for translocation of both Tir and Map	EPEC/STEC	100.00	471 / 471	LT903847
*chuA*	*E.coli* hemeutilization protein A gene	ExPEC	99.65	1983 / 1983	LT827011
*cif*	Type III secreted effector	EPEC/STEC	100.00	849 / 849	AF497476
*csgA*	Major curlin subunit	Several	99.56	459 / 459	CP023388
*csgB*	Minor curlin subunit	Several	99.56	456 / 456	CP027060
*csgE*	Curli production assembly/transport component	Several	98.70	386 / 390	NC_011750
*csgF*	Curli production assembly/transport component	Several	98.08	417 / 417	NC_011750
*csgG*	Curli production assembly/transport component	Several	97.72	834 / 834	CP003034
*cvaA*	Colicin V secretion protein gene	Several	100.00	1242 / 1242	GG773553
Death on curing RelE/ParE family toxin gene	Component of Toxin-antitoxin (TA) system	Several	100.00	272 / 276	CP023388
*eae* (subtype Beta2)	Intimin - Necessary for the production of attaching and effacing lesions on tissue culture cells	EPEC/STEC	100.00	2820 / 2820	AB647493
*ecpA*	Common pilus major fimbrillin subunit	Several	98.47	588 / 588	BA000007
*ecpD*	Fimbria adhesin of the *E.coli* common pilus	Several	99.64	1644 / 1644	CP019777
*elfC*	Putative fimbrial usher protein	Several	99.73	2595 / 2595	CP021288
*entA*	Enterobactin biosynthesis gene	Several	96.12	747 / 747	CP027060
*entE*	Enterobactin biosynthesis gene	Several	95.65	1611 / 1611	CP027060
*entH*	Enterobactin biosynthesis gene	Several	94.93	414 / 414	CP027060
*escC*	Outer membrane secretin	EPEC/STEC	91.68	1539 / 1539	AP010958
*escD*	Type III secretion system inner membrane ring protein	EPEC/STEC	99.10	1221 / 1221	BA000007
*escF*	Type III secretion system needle major subunit	EPEC/STEC	100.00	222 / 222	NC_002695
*escJ*	Required for the formation of the type III Secretion Apparatus	EPEC/STEC	91.62	573 / 573	AP010958
*escN*	Type III secretion ATPase	EPEC/STEC	100.00	1341 / 1341	BA000007
*escR*	Type III secretion system export apparatus protein gene	EPEC/STEC	99.69	654 / 654	BA000007
*escS*	Type III secretion system export apparatus protein gene	EPEC/STEC	100.00	270 / 270	BA000007
*escT*	Type III secretion system export apparatus protein gene	EPEC/STEC	99.61	777 / 777	BA000007
*escU*	Type III secretion system LEE export apparatus switch protein gene	EPEC/STEC	96.15	1038 / 1038	AP010958
*escV*	Translocase of the type III secretion system	EPEC/STEC	99.70	2028 / 2028	BA000007
*espA*	Type III secretions system gene	EPEC/STEC	100.00	573 / 573	AJ225016
*espG*	Type III secretion system effector, which localize to the Golgi apparatus and disrupt its architecture	EPEC/STEC	98.41	1197 / 1197	BA000007
*etgA*	Lytic transglycosylase	EPEC/STEC	100.00	459 / 459	FM986650
*fdeC*	Mediates *E. coli* adhesion to mammalian cells and extracellular matrix	ExPEC/STEC	97.86	4251 / 4251	CP019777
*sfaH*	S-fimbrial protein subunit gene	ExPEC	98.56	903 / 903	KT444704
*flgD*	Flagellar basal body rod modification protein gene	Several	96.55	696 / 696	CP027060
*flgM*	Negative regulator of flagellin synthesis	Several	98.97	290 / 294	CP028192
*fmlA*	Major F9-fimbrial subunit	ExPEC/IPEC	96.81	564 / 564	BA000007
*fyuA*	Ferric yersiniabactin uptake receptor	ExPEC	99.51	2022 / 2022	CP016828
*gad (1)*	Glutamate decarboxylase gene	Several	99.64	1401 / 1401	CP001671
*gad (2)*	Glutamate decarboxylase gene	Several	99.79	1401 / 1401	FM180568
*grlA*	Global regulator of LEE activator	EPEC/STEC	97.56	409 / 414	AP010958
*gtrA*	Type IV O-antigen modification gene (*Shigella flexneri*)	Unknown	90.08	363 / 363	AF288197
*hbp*	Hemoglobin-binding protease hbp autotransporter gene	ExPEC	99.95	4131 / 4131	CP009072
*hlyIII*	Gene encoding inner membrane protein, hemolysin III family	ExPEC/IPEC	98.41	690 / 690	CP003034
*ibeA*	Invasion protein gene	NMEC/APEC/AIEC	98.61	1371 / 1371	CP001855
*ibeB*	Invasion protein gene	Several	98.55	1383 / 1383	AF094824
*ibeC/yijP/cptA*	Invasion protein gene	Several	99.77	1734 / 1734	CP019777
*irp1*	HMWP1 nonribosomal peptide/polyketide synthase	ExPEC	99.65	9492 / 9492	CU928163
*irp2*	HMWP2 Yersiniabactin biosynthetic protein	ExPEC	98.85	6106 / 6108	CP006834
*ler*	Negative autoregulator of the LEE1 operon	EPEC/STEC	99.49	390 / 390	BA000007
*malX*	*Escherichia coli* pathogenicity island-marker	ExPEC	98.61	1581 / 1581	AF003742
*MAP*	LEE effector protein gene	EPEC/STEC	97.06	612 / 612	LC053401
MBL-fold metallo hydrolase gene	Putative phylogroup B2 specific marker	ExPEC	99.52	1044 / 1044	CP023388
*mdtH*	Multidrug resistance protein gene	Several	99.83	1209 / 1209	CP019777
*mpc*	Type III secretion system regulator gene	EPEC/STEC	92.09	354 / 354	AP010953
*mviM*	Putative virulence factor	Several	98.70	924 / 924	CU928164
*nleA* ^a^	Non-LEE encoded effector A	EPEC/STEC	99.84	1239 / 1239	AB303062
*nleB*	Non-LEE encoded effector B	EPEC/STEC	100.00	981 / 981	AB303062
*nleC*-like gene	T3SS secreted effector NleC-like protein gene	EPEC/STEC	100.00	264 / 264	CYEL01000033
*nleG*	Non-LEE encoded effector G	EPEC/STEC	100.00	576 / 576	AB303062
*nleH*	Non-LEE encoded effector H	EPEC/STEC	99.75	812 / 812	AP010958
*usp*/putative colicin	Uropathogenic specific protein gene	UPEC	97.14	1782 / 1782	CU651637
*sepL*	Secretion switching protein gene	EPEC/STEC	94.93	1046 / 1056	BA000007
*sepQ*	T3SS structure protein	EPEC/STEC	95.53	918 / 918	CP003109
*sinH*	Intimin-like inverse autotransporter	ExPEC	100.00	2178 / 2178	NZ_NMHI01000013
*stcD*	Putative fimbrial-like adhesin protein gene	IPEC	99.71	1035 / 1035	NC_018658
*stfD*	Fimbrial protein gene	Unknown	100.00	753 / 753	LOFW01000008
*tir*	Translocated intimin receptor protein gene	EPEC/STEC	99.88	1650 / 1650	DQ206455
*xhlA*	*Xenorhabdus nematophila* haemolysin	Unknown	99.73	372 / 372	LDCR01000046
*ybtA*	Yersiniabactin transcriptional regulator	ExPEC	99.79	960 / 960	CP028714
*ydeR*	Fimbrial-like protein gene	Several	98.41	504 / 504	CU928163
*yfcV*	Major subunit of a putative chaperone-usher fimbria	ExPEC	97.18	567 / 567	NC_011750

^a^Frameshifted

The *alkB* gene coding for the alkylated DNA repair protein AlkB has an internal frameshift, and is probably inactive in FHI_NMBU_03. Several loci pertaining to fimbrial structures were found and noteworthy are genes related to K88-fimbria, 987P-fimbria and colonization factor antigen I fimbriae (CFA/I), which are all associated with ETEC strains. FHI_NMBU_03 is also positive for the YghJ protein gene, also known as SslE (Secreted and surface associated lipoprotein), which is a cell surface associated and secreted lipoprotein harbouring M60 metalloprotease domain [28].

A previously reported insertion of unknown origin with a base composition suggestive of horizontal gene transfer in a genetic region between *mutS* and *rpoS*, associated with phylogroup B2 and uropathogens [29] is additionally present. This region has later been named the *o454-nlpD* region [30].

## Discussion

Clinical microbiological laboratories and reference laboratories rely increasingly on genetic testing of faeces to identify possible pathogenic microbes. For enteric bacteria, a widely used practice is to perform PCR or real-time PCR assays, or other amplification methodology, to detect specific genes used for pathogen identification. For *E. coli*, PCR on faecal isolates [13] is used to detect the well-recognized IPEC pathovars EPEC, STEC, ETEC, EAEC and EIEC [31]. These pathovars all have genetic targets used for identification and classification. The most common genetic targets are the *eae* and *bfp* genes for EPEC, *stx1* and *stx2* genes for STEC, genes encoding the thermostable (ST) and thermolabile (LT) toxins for ETEC, the *aggR* gene for EAEC, and the *ipaH* gene for EIEC. These targets are also candidate targets for automatic pathogen identification systems, especially in a culture-independent diagnostic tests (CIDTs) workflow. The results from these assays will be a classification of the *E. coli* isolates into one of the recognized pathovars or, in case of no target amplification, a classification as a non-enteropathogenic or commensal strain.

In the present study, we looked at a wider range of virulence factors in faecal *E. coli* isolates submitted to the Reference Laboratory for Enteropathogenic Bacteria at the Norwegian Institute of Public Health (NIPH). We especially searched for known ExPEC VAGs as in recent years a heighten interest in the frequency of ExPEC strains in the human gut has emerged, however there are few studies examining the selection of VAGs used in the present study.

One surprising finding in our study was the high frequency of *E. coli* strains (64.3%) with a combination of recognized IPEC and ExPEC VAGs. There are limited data on how common these IPEC/ExPEC hybrid strains are. In a study of 265 *E. coli* isolates from hospital inpatients and outpatients with UTIs, 10.6% of isolates harboured at least one IPEC virulence factor [32]. In previous studies of human faecal isolates, the *E. coli* strains are separately designated as IPEC or as commensal strains harbouring ExPEC VAGs, thus it is unclear how high of a percentage may be IPEC/ExPEC combinatory strains. The IPEC/ExPEC combination was especially high among the aEPEC strains (91.8%).

One notable finding was that 13 out of 14 (92.9%) *ibeA* positive isolates was an EPEC strains of phylogenetic group B2. Thus, *ibeA* carriage in faeces seems to be associated with a distinct group of IPEC strains in our material. The *ibeA* gene is a known virulence factor of *E. coli* strains responsible for neonatal meningitis in humans (NMEC) by contributing to the invasion of brain microvascular endothelial cells (BMEC) [33]. It has also been described that *ibeA* plays an important role in the invasion of intestinal epithelial cells, as the absence of *ibeA* accounted for a reduction in invasion of ca. 67% compared to wild type in experiments with the adherent-invasive *E. coli* (AIEC) strain NRG857c and an *ibeA* deletion mutant strain (NRG857cΔ*ibeA*) [34]. Furthermore, *ibeA* was present in the genome of 26% of pathogenic isolates from chicken (APEC), but absent from the genome of non-pathogenic isolates of avian origin [35]. The *ibeA* gene was positively linked to the pathogenicity of the APEC strains, and it was additionally shown that *ibeA* was involved in the invasion of human BMEC by the APEC strain BEN 2908 [35].

An interesting observation was the high number of strains harbouring genes coding for the trimeric autotransporter proteins (TAAs) EhaA and EhaG. Especially finding the *ehaG* gene in 48% of the strains with one or more ExPEC VAGs and no IPEC VAGs, since EhaG mediates specific adhesion to colorectal epithelial cells [36]. This indicates that 48% of our isolates carrying solely ExPEC VAGs may have the capacity to adhere to colorectal epithelial cells in humans. Both *ehaA* and *ehaG* are most prevalent in the phylogenetic groups B1 and D, while a difference between *ehaA* and *ehaG* was observed in phylogenetic group A where *ehaA* was not detected but *ehaG* was present in 34% of the isolates. The distribution pattern of *ehaA* and *ehaG* was in the same range as results from a study by Zude et al. 2014 [37], with the exception of phylogenetic group B2 where Zude et al. 2014 report that 21.9% of the strains carry the *ehaG* gene, while in the present study 7.1% of the B2 strains were positive for *ehaG*. EhaG is localized at the bacterial cell surface and, in addition to colorectal epithelial cell adhesion, promotes cell aggregation, biofilm formation, and adherence to a range of extracellular matrix (ECM) proteins [36]. TAAs are regarded as important virulence factors of many Gram-negative bacterial pathogens. We are aware that our PCR-based phylogrouping results may show minor differences from the 2013 Clermont method [38]. Non-IPEC strains are not stored at NIPH thus a re-typing of all strains using the 2013 Clermont method on all strains in this study is not possible, however the findings and conclusions are valid, and in future our phylogrouping will be sequenced-based e.g. by using online tools [39].

The fully sequenced FHI_NMBU_03 phylogroup B2 strain (with plasmid) from this study shows hallmarks of ExPEC pathovars UPEC, APEC, NMEC and the IPEC pathovar aEPEC with some VAGs related to ETEC (K88-, 987P- and CFA/I- fimbrial genes), thus it constitutes a truly pathovar-hybrid strain (Additional file 3). The *eae* gene alone will classify it as an aEPEC by most molecular diagnostics tests.

It was previously reported that YghJ caused extensive haemorrhage in mouse ileum in a dose dependent manner and it was suggested that YghJ could be a virulence factor of enteric pathogens associated with haemorrhagic diarrhoea [28]. A recent study additionally showed that the YghJ protein from a neonatal septicaemic *E. coli* altered cellular morphology of various cell lines and triggered the induction of several proinflammatory cytokines, which are attributed as one of the key mediators in the pathogenesis of sepsis [40].

Several factors classify this strain as UPEC (e.g. *usp*, *fyuA*, *sfaS,* the *pap* fimbrial operon, *chuA* and *yfcV*). It has previously been reported that any two of *yfcV*, *vat*, or *chuA* along with *fyuA* could be used to differentiate UPEC from diarrheagenic *E. coli* (DEC), human commensal, or animal commensal isolates. However, to differentiate UPEC from APEC, *vat, fyuA*, and *yfcV* together are necessary, where the presence of the putative fimbrial subunit gene *yfcV* is highly predictive of UPEC, increasing the odds of a strain being UPEC by 99.5-fold [41].

The fimH90 subtype was also an interesting finding as it appears to be rare among *E. coli* strains and was not found among 243 draft genomes of *E. coli* isolates in a study using the CGE FimTyper Web tool [42]. However, BLAST searches found an identical *fimH* gene in a sequence scaffold from a human aEPEC strain (702898_aEPEC) isolated in Pakistan (GenBank: CYBW01000017.1). The CGE FimTyper confirmed this *fimH* gene to also be of subtype fimH90.

The comparison of sequence data with PCR typing revealed PCR positive results for *tsh* and *vat* while sequencing showed the presence of the highly related *hbp* gene on the chromosome and a putative related autotransporter on the virulence plasmid (locus tag: BXO92_24355). The PCR results can be explained by the similarity of the intended target genes, and the considerable confusion in GenBank submitted sequences on the correct nomenclature. The Tsh and Hbp proteins differ by only two amino acid residues. In addition, Vat and Tsh/Hbp are 77.5% identical in amino acids.

The plasmid located putative autotransporter protein (protein id: PRJNA362852:BXO92_24355) show 43.7% AA identity and 56.6% AA similarity to Tsh. RAST annotates this protein as EspC, while BASys annotates it as Hbp.

The number of GIs and integrated prophages indicate that FHI_NMBU_03 has obtained a high number of virulence factors by horizontal gene transfer and this may have been facilitated by a defect in the DNA-repair system with a frameshifted *alkB* gene. It is known that AlkB relevant lesions appear to represent strong blocks to replication, but these blocks can be bypassed by error-prone translesion DNA polymerases as a part of the SOS-system, leading to mutagenesis [43].

The *o454-nlpD* region was shown to consist of several genetic patterns, where pattern III (the FHI_NMBU_03 sequence contains pattern III) had significant associations with phylogenetic group B2 strains, representing the most virulent members of the ExPEC group. This *o454-nlpD* region pattern was proposed as a tool to identify highly extraintestinal virulent strains among a mixed population of *E. coli* [30].

Strains closely related to FHI_NMBU_03 may have caused disease in Norway for an extended period of time as nine aEPEC intimin *eae-*β2 carrying B2 strains of sequence type ST28 was previously detected among 56 aEPEC isolates from faecal specimens from children < 5 years old in Norway (five strains were from community-acquired diarrhoea samples) [44]. All nine strains where shown by microarray analysis to contain the *ibeA*, *malX* and *usp* genes as FHI_NMBU_03.

The high frequency of strains with combined IPEC/ExPEC VAGs found in this study is worrisome as they might be capable of causing both intestinal- and extraintestinal disease. One scenario could be a general weakening of the immune system caused by ongoing intestinal disease, thereby creating an opportunity for spread of bacteria with ExPEC VAGs to other anatomical sites where the ExPEC VAGs may contribute to severe extraintestinal disease.

## Conclusion

We report that a high frequency (> 93%) of routinely submitted faecal *E. coli* strains from Norwegian hospitals, previously characterized as IPEC, also harbour ExPEC virulence factors. Traditionally IPEC is regarded as a diarrhoeagenic pathogen with a set of virulence genes that is absent in ExPEC strains e.g. UPEC. This very high frequency of combined IPEC/ExPEC was an unexpected finding warranting further studies, as they may provide a rich source of opportunistic extraintestinal infections. WGS of one selected strain confirmed the pathovar-hybrid nature and revealed a genome heavily influenced by horizontal gene transfer (HGT). Sequence complex ST28 has previously been assigned to a hybrid group that was named “phylogroup ABD” [45], which supports our finding of the hybrid nature for strain FHI_NMBU_03.

## Additional files

Additional file 1: PCR primers used in study. Sequences of all PCR-primers used in this study, with references. (DOCX 18 kb)

Additional file 2: The Excel sheet contains VAGs PCR, Phylogenetic PCR and MLVA results for all *E. coli* strains included in this study. - PCR positive amplicons are listed as well as the MLVA profile and the results from the phylogenetic group PCR. (XLSX 21 kb)

Additional file 3: FHI-NMBU-03 SNPtree03 slanted. The image shows results from comparing the genome of FHI-NMBU-03 with a selection of *E. coli* whole genomes with *E. coli* K-12 MG1655 as reference. The SNP based phylogenetic tree was constructed using CSI Phylogeny 1.4 (https://cge.cbs.dtu.dk/services/CSIPhylogeny/). (PDF 10 kb)

## Abbreviations

aEPEC: Atypical enteropathogenic *Escherichia coli*
AIEC: Adherent-invasive *Escherichia coli*
APEC: Avian pathogenic *Escherichia coli*
BMEC: Brain microvascular endothelial cells
CGE: Center for Genomic Epidemiology
cnf: Cytotoxic necrotising factor
DEC: Diarrheagenic *Escherichia coli*
*E. coli*: *Escherichia coli*
EAEC: Enteroaggregative *Escherichia coli*
EIEC: Enteroinvasive *Escherichia coli*
EnPEC: Endometrial pathogenic *Escherichia coli*
EPEC: Enteropathogenic *Escherichia coli*
ETEC: Enterotoxigenic *Escherichia coli*
ExPEC: Extraintestinal pathogenic *Escherichia coli*
GIs: Genomic islands
HGT: Horizontal gene transfer
IPEC: Intestinal pathogenic *Escherichia coli*
LEE: The locus of enterocyte effacement
MLST: Multilocus sequence typing
MLVA: Multiple Locus Variable-Number Tandem-Repeat Analysis
NIPH: Norwegian Institute of Public Health
NMEC: Neonatal meningitis *Escherichia coli*
NTEC: Necrotoxin producing *Escherichia coli*
ORFs: Open reading frames
SePEC: Septicaemic *Escherichia coli*
STEC: Shiga toxin producing *Escherichia coli*
TAAs: Trimeric autotransporter proteins
tEPEC: Typical enteropathogenic *Escherichia coli*
UPEC: Uropathogenic *Escherichia coli*
UTI: Urinary tract infection
VAGs: Virulence factors
WGS: Whole genome sequencing
