# Supplementary material for: High frequency of hybrid Escherichia coli strains with combined Intestinal Pathogenic Escherichia coli (IPEC) and Extraintestinal Pathogenic Escherichia coli (ExPEC) virulence factors isolated from human faecal samples
Source: BMC Infect Dis. 2018 Nov 1;18:544. doi: 10.1186/s12879-018-3449-2 (PMC6267907; doi:10.1186/s12879-018-3449-2)
Supplement: Supplementary file 1 — PCR primers used in study. Sequences of all PCR-primers used in this study, with references. (DOCX 18 kb) [file 12879_2018_3449_MOESM1_ESM.docx]

**PCR primers used in study.**

ExPEC VAGS with *ehaA* and *ehaG*.

Name Dye, sequence and amplicon size

| CNF1F | 5’ – (6FAM) - GGT CAG GGC CCA CAG TCA A – 3’ | 406bp | This study |
| --- | --- | --- | --- |
| CNF1R | 5’ – TAG CGG CTT CAA AAT ACG GAT AG – 3’ |  |  |
| CNF3F | 5’ – (VIC) - AAG GCC AGT GAA AAT AAG GTG ATA – 3’ | 429bp | This study |
| CNF3R | 5’ – TTC CGG AGA CGA TGC CAG TT – 3’ |  |  |
| IUTAF | 5’ – (NED) - AAT CCC GGC AGC TTC AGT TG – 3’ | 491bp | This study |
| IUTAR | 5’ – ATA GCG CCC GGT GGT GTA AAT CT – 3’ |  |  |
| CNF2F | 5’ – (PET) - GCG GCA AGC AGA CGA CA – 3’ | 506bp | This study |
| CNF2R | 5’ – GAG CCG AGC GAG GAT GC – 3’ |  |  |
| IBEAF | 5’ – (6FAM) - GGG CGG ATG AAG AAA ATA AAA ACT – 3’ | 553bp | This study |
| IBEAR | 5’ – GCC CGT CCG CAA ACC AA – 3’ |  |  |
| SITAF | 5’ – (VIC) - CGC GTG CGA GAT AAG AAA AAG – 3’ | 557bp | This study |
| SITAR | 5’ – GGG GGC CTC GCA CTC AC – 3’ |  |  |
| IUCDF | 5’ – (6FAM) - GGA GTG GCC CGA AAG AGA ACA ACA – 3’ | 160bp | This study |
| IUCDR | 5’ – AGG GCG GGC GGC ATA CTG A – 3’ |  |  |
| iss-F | 5’ – (VIC) - GTT ATT TTC TGC CGC TCT GG – 3’ | 227bp | [Fricke et al., 2009](https://www.ncbi.nlm.nih.gov/pmc/articles/PMC2747853/bin/supp_75_18_5963__index.html) |
| iss-R | 5’ – AAC CGA GCA ATC CAT TTA CG – 3’ |  |  |
| TRATF | 5’ – (NED) - GCC AGC GAA CGC ACG GTA TTT – 3’ | 428bp | This study |
| TRATR | 5’ – TGA CGA AGA GCG GCA ACA TTA TCC – 3’ |  |  |
| IROCF | 5’ – (PET) - TAT GCC GGG ATG CTC TCA CTC TGG – 3’ | 450bp | This study |
| IROCR | 5’ – CAC AAT CTG CCG CGA CTG CCT ATC – 3’ |  |  |
| SATF | 5’ – (6FAM) - GGC GCG GGC TGT GAC TTT C – 3’ | 461bp | This study |
| SATR | 5’ – AAC TCG ATG GCC TTG ACC TCT TC – 3’ |  |  |
| PAPCF | 5’ – (VIC) - GCC CGT TCC CCA GCG ATT TGT – 3’ | 483bp | This study |
| PAPCR | 5’ – CAG GGT GTG GCG GTT GGA TTG G – 3’ |  |  |
| EHAAF | 5’ – (NED) - CGC GGG CGA TAA CAG CAA CC – 3’ | 485bp | This study |
| EHAAR | 5’ – AGT CAT ACG CCC CCG CAA CGA TAC – 3’ |  |  |
| tshF | 5’ – (NED) - GCA GGG GCA TCC GGT TAT CCA T – 3’ | 410bp | This study |
| tshR | 5’ – TGT TCG CCT GTA TTC CGC CAT TGA – 3’ |  |  |
| gimBF | 5’ – (6FAM) - TCA GGC AAG AAA TGG TAA GGA CA – 3’ | 464bp | This study |
| gimBR | 5’ – AAG CGG CAT GAG GGG TAA TAG – 3’ |  |  |
| etsAF | 5’ – (VIC) - ATT CGA TGT GCC TAA CCC AG – 3’ | 286bp | This study |
| etsAR | 5’ – CCT TTC TTC GGG CTC TTT CT – 3’ |  |  |
| kpsSF | 5’ – (PET) - CCA TAA ATC GTT TTC CCC CT – 3’ | 415bp | This study |
| kpsSR | 5’ – GTT AAT CGT CAC CAC CGC TT – 3’ |  |  |
| sfaSF | 5’ – (6FAM) - TGG CCA CCG GTC TTA TTA AC – 3’ | 210bp | This study |
| sfaSR | 5’ – ATA TTC TGG CAT CCG GTG AG – 3’ |  |  |
| tosA-F | 5’ – (PET)-AGG GGG ATG CTA CTG ACA ATA ACG – 3’ | 401bp | This study |
| tosA-R | 5’ – ATC TCC CGC CCC CAG TGA AAT AG – 3’ |  |  |
| vat-F | 5’ – (6FAM)-CCC GGG GTT GCT TTA TTT – 3’ | 413bp | This study |
| vat-R | 5’ – GGG GGT TCT GGG ATG TTA – 3’ |  |  |
| fbpB-F | 5’ – (NED)-GTA GCC GGA GCG CCC CAT CAT – 3’ | 436bp | This study |
| fbpB-R | 5’ – CGC CGC CAT CGT GCT GTT TAT C – 3’ |  |  |
| fyuA-F | 5’ – (VIC)-GAG CGG CCC CAT TCA GGA – 3’ | 510bp | This study |
| fyuA-R | 5’ – TGC CGG TAC AGC CCA AAC AC – 3’ |  |  |
| EhaG-F | 5’ – (6FAM)-CGC GCA GGG TAA AGA TAG C – 3’ | 549bp | This study |
| EhaG-R | 5’ – ACT GCC GAT TCA CCG TTG TA – 3’ |  |  |

IPEC VAGs with control.

Name Dye, sequence and amplicon size

| stx1-F | 5’ – (HEX)- AAATCGCCATTCGTTGACTACTTCT – 3’ | 370bp | [Brian et al., 1992](https://www.sciencedirect.com/science/article/pii/S0167701206002776?via%3Dihub#bib6) |
| --- | --- | --- | --- |
| stx1-R | 5’ – TGCCATTCTGGCAACTCGCGATGCA – 3’ |  |  |
| stx2-F | 5’ – (6FAM) – CAGTCGTCACTCACTGGTTTCATCA – 3’ | 283bp | [Brian et al., 1992](https://www.sciencedirect.com/science/article/pii/S0167701206002776?via%3Dihub#bib6) |
| stx2-R | 5’ – GGATATTCTCCCCACTCTGACACC – 3’ |  |  |
| eae-F | 5’ – (TET) – TCAATGCAGTTCCGTTATCAGTT – 3’ | 482bp | [Vidal et al., 2004](https://www.sciencedirect.com/science/article/pii/S0167701206002776?via%3Dihub#bib39) |
| eae-R | 5’ – GTAAAGTCCGTTACCCCAACCTG – 3’ |  |  |
| STIb-F | 5’ – (TET) – ATTTTTCTTTCTGTATTGTCTT – 3’ | 190bp | [Lopez-Saucedo et al., 2003](https://www.sciencedirect.com/science/article/pii/S0167701206002776?via%3Dihub#bib23) |
| STIb-R | 5’ – CACCCGGTACAAGCAGGATT – 3’ |  |  |
| STIa-F | 5’ – TCTTTCCCCTCTTTTAGTCAG – 3’ | 166bp | [Woodward et al., 1991](https://doi.org/10.1016/0378-1135(92)90083-6) |
| STIa-R | 5’ – ACAGGCAGGATTACAACAAAG – 3’ |  |  |
| LTI-F | 5’ – (TET) - TCTCTATGTGCATACGGAGC – 3’ | 322bp | [Rappelli et al., 2001](https://www.sciencedirect.com/science/article/pii/S0167701206002776?via%3Dihub#bib29) |
| LTI-R | 5’ – CCATACTGATTGCCGCAAT – 3’ |  |  |
| ipaH-F | 5’ – (HEX) – GTTCCTTGACCGCCTTTCCGATACCGTC – 3’ | 619bp | [Toma et al., 2003](https://www.sciencedirect.com/science/article/pii/S0167701206002776?via%3Dihub#bib37) |
| ipaH-R | 5’ – GCCGGTCAGCCACCCTCTGAGAGTAC – 3’ |  |  |
| aggR-F | 5’ – (HEX) – GTATACACAAAAGAAGGAAGC – 3’ | 254bp | [Toma et al., 2003](https://www.sciencedirect.com/science/article/pii/S0167701206002776?via%3Dihub#bib37) |
| aggR-R | 5’ – ACAGAATCGTCAGCATCAGC – 3’ |  |  |
| ehxA-F | 5’ – AAACAACGGGAAGGAGAG – 3’ | 233bp | [Chahed et al., 2006](https://www.ncbi.nlm.nih.gov/pmc/articles/PMC3370565/#B20) |
| ehxA-R | 5’ – ACAACATCCAGCCCA – 3’ |  |  |
| bfp-F | 5’ – GATAAAACTGATACTGGGCAGC – 3’ | 826bp | [Müller et al., 2006](https://dx.doi.org/10.1128%2FJCM.00895-06) |
| bfp-R | 5’ – AGTGACTGTTCGGGAAGCAC – 3’ |  |  |
| rrs-F | 5’ – (6FAM) – CCCCCTGGACGAAGACTGAC – 3’ | 401bp | [Wang et al., 2002](https://www.sciencedirect.com/science/article/pii/S0167701206002776?via%3Dihub#bib40) |
| rrs-R | 5’ – ACCGCTGGCAACAAAGGATA – 3’ |  |  |
