# Supplementary figures and images for: High frequency of hybrid Escherichia coli strains with combined Intestinal Pathogenic Escherichia coli (IPEC) and Extraintestinal Pathogenic Escherichia coli (ExPEC) virulence factors isolated from human faecal samples
Source: BMC Infect Dis. 2018 Nov 1;18:544. doi: 10.1186/s12879-018-3449-2 (PMC6267907; doi:10.1186/s12879-018-3449-2)

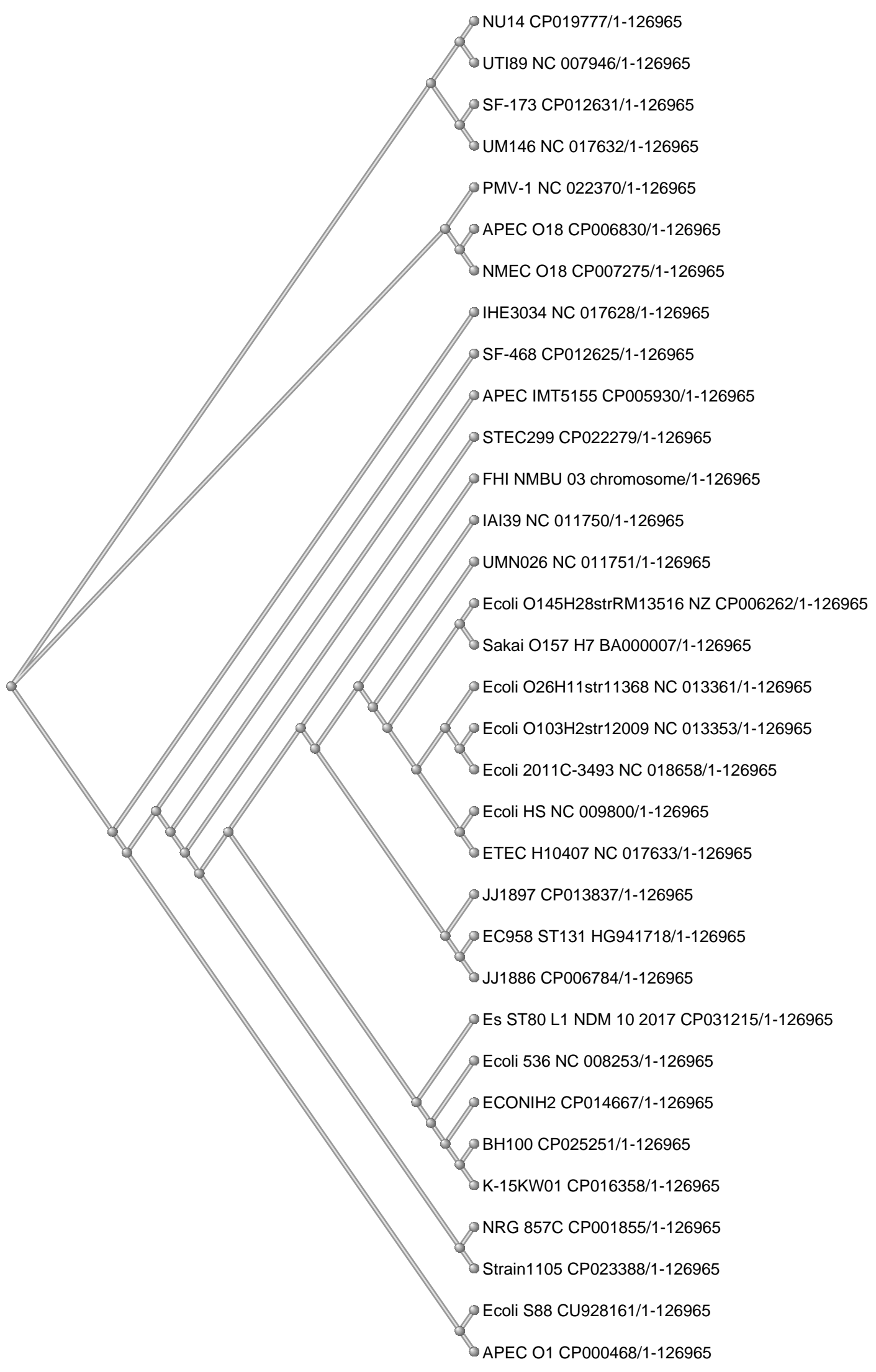

Supplement: Supplementary file 3 — FHI-NMBU-03 SNPtree03 slanted. The image shows results from comparing the genome of FHI-NMBU-03 with a selection of E. coli whole genomes with E. coli K-12 MG1655 as reference. The SNP based phylogenetic tree was constructed using CSI Phylogeny 1.4 (https://cge.cbs.dtu.dk/services/CSIPhylogeny/). (PDF 10 kb) [file 12879_2018_3449_MOESM3_ESM.pdf]
